# Prevalence and Determinants of Bad Sleep Perception among Italian Children and Adolescents

**DOI:** 10.3390/ijerph17249363

**Published:** 2020-12-14

**Authors:** Serena Malloggi, Francesca Conte, Giorgio Gronchi, Gianluca Ficca, Fiorenza Giganti

**Affiliations:** 1 Department NEUROFARBA, University of Firenze, 50135 Firenze, Italy; serena.malloggi@unifi.it (S.M.); giorgio.gronchi@unifi.it (G.G.); 2 Department of Psychology, University of Campania L. Vanvitelli, 81100 Caserta, Italy; francesca.conte@unicampania.it (F.C.); gianluca.ficca@unicampania.it (G.F.)

**Keywords:** children, adolescents, subjective sleep quality

## Abstract

Although sleep problems at young ages are well investigated, the prevalence of bad sleepers and the determinants of sleep quality perception remain unexplored in these populations. For this purpose, we addressed these issues in a sample of children (*n* = 307), preadolescents (*n* = 717), and adolescents (*n* = 406) who completed the School Sleep Habits Survey, addressing sleep quality perception, sleep habits, sleep features, daytime behavior and sleep disturbances, circadian preference, and dreaming. The sample was split in “good sleepers” and “bad sleepers”, based on the answer to the question item assessing overall subjective sleep quality. Being a bad sleeper was reported by 11.7% of the sample, with significant between-groups differences (children: 8.3%; preadolescents: 11.3%; adolescents: 15.3%; *p* = 0.01). At all ages, relative to good sleepers, bad sleepers showed higher eveningness, sleepiness, and depression, longer sleep latency, more frequent insufficient sleep, nocturnal awakenings, sleep–wake behavioral problems, and unpleasant dreams (all *p*’s ≤ 0.01). Sleep quality perception was predicted: in children, by depressed mood, eveningness, and unpleasant dreams (all *p*’s ≤ 0.01); in preadolescents, by sleep latency, awakening frequency, depressed mood, sufficiency of sleep, and unpleasant dreams (all *p*’s < 0.01); in adolescents, by awakening frequency, depressed mood, and sufficiency of sleep (all *p*’s < 0.001). In children, bad subjective sleep quality appears to be mainly determined by daytime psychological features, for example, depressed mood, whereas at later ages, sleep characteristics, such as frequent awakenings, add to the former determinants. This could depend on (a) the appearance, with increasing age, of objective sleep modifications and (b) a greater attention paid by adolescents to their sleep characteristics.

## 1. Introduction

During childhood and adolescence, sleep undergoes significant changes that include modifications of amount, distribution, and characteristics of sleep architecture parameters [1,2,3,4]. These modifications are often associated with sleep disruptions. Indeed, numerous surveys report, in young populations, a high prevalence of sleep problems, such as night awakenings [5,6], nightmares [7], nocturnal enuresis [8,9], sleep onset delay [10,11], and sleep restriction [12]. Also, the consequences of poor sleep quality on school performance in children and adolescents have been widely explored (see [13] for a meta-analytic review).

Despite this ample literature on patterns and problems of sleep in these young populations, a very limited number of studies have explored their perceived sleep quality. In a sample of 449 Dutch children aged between 9 and 14 years, Meijer and colleagues [14] reported mediocre or bad quality of sleep in 22.4% of the subjects, but in this study sleep quality perception was solely considered as one of the factors (the others being objective sleep features) contributing to a global “quality of sleep” score.

Other studies, performed on Italian samples, mainly focused on sleep habits, circadian preference [15,16,17], ethnic and sociocultural influences [18], and the presence of specific sleep problems (e.g., insomnia and obstructive sleep apnea syndrome [19,20,21]) rather than subjective sleep quality perception. Therefore, there are very few studies based on representative samples of Italian children and adolescents on self-reported sleep impairments.

Available data on Italian students point to relevant changes in sleep habits and features that occur from childhood to adolescence, namely, delayed bedtime, lower sleep duration, discrepancies in sleep–wake schedules between schooldays and weekends, and a trend to eveningness increasing with age [15,16,17,18].

In Italian children and adolescents, poor sleep has been related both to eveningness [15,16] and to bad sleep hygiene practices [22]. Actually, the prevalence of poor sleep perception has been addressed only in adolescents: poor subjective sleep quality was reported by 19% of a sample of Italian students [17], and complaints indicative of persisting bad sleep were found in 16.5% of adolescents belonging to a specific area of Northern Italy [22].

The lack of literature on sleep quality perception in younger populations appears all the more surprising when considering the relevance of subjective sleep quality for human well-being and daily functioning [23,24,25,26,27], as well as the fact that sleep quality perception is a key factor in diagnosing sleep disturbance [28] and evaluating treatment outcomes (e.g., [29,30]). In addition, subjective sleep quality measures in young subjects appear to show a stronger relationship with school performance than objective measurements [13], especially when sleep quality is evaluated using self- rather than parental reports. Indeed, parental awareness of their children’s sleep (both children and adolescents) seems to be rather limited [13,31].

Another related issue refers to the determinants of sleep satisfaction at these younger ages. In the adult, a wide literature exists exploring the relationships of subjective sleep quality judgments with sleep measures (e.g., [32,33,34,35,36,37,38]). Numerous objectively measured sleep variables have been shown to relate to sleep quality perception: total sleep time [39,40], slow wave sleep duration [33,38,39], wake after sleep onset time [39,41], sleep efficiency ([33,39,42], number of night awakenings [43,44,45], sleep stability (arousals and state transitions frequency, [45]), and sleep organization (number of sleep cycles and time spent in cycles, [45]). As for self-reported sleep features, their relationship to subjective sleep quality has been more seldom investigated, although it has been observed that self-reported measures are stronger predictors of perceived sleep quality than actigraphy-based sleep measures [46]. According to Akerstedt and colleagues [32,42], subjective sleep quality is mainly predicted by subjective calmness of sleep, ease of falling asleep, and ability to sleep throughout the allotted time, while Goelema et al. [46] found that self-reported number of awakenings and total sleep time were the best predictors. Other subjective factors, such as general good health and stress before bedtime, have also been reported as linked to sleep quality perception [47].

In contrast to this broad literature concerning adults, factors underlying sleep satisfaction judgments in young and elderly individuals have been poorly explored. Moreover, it is not known whether these determinants might exhibit age-related differences. To our knowledge, determinants of sleep perception at young ages have been retrospectively investigated in one recent study conducted on a sample of Dutch children from lower socioeconomic neighborhoods [48]. The children were directly asked to report potential reasons for their inadequate sleep and their relative relevance [48]. Nightmare experiences and presence of illnesses appeared as the main subjective determinants of poor sleep perception [48]. As for the aged population, Zilli and colleagues [49] found that elderly individuals’ sleep satisfaction is mainly based on sleep latency and duration rather than continuity (i.e., they maintain a perception of good sleep despite a high number of nocturnal awakenings). Interestingly, the authors also observed that subjective determinants of sleep satisfaction differ between adults and elderly people: freshness after awakening appears relevant for good sleep perception in aged individuals, whereas frequency of awakenings is more salient in younger ones [49].

In continuity with the latter studies, our work aims to address the relevant gaps in literature on subjective sleep quality over the life span, by studying a sample of Italian children, preadolescents, and adolescents. In particular, by addressing sleep satisfaction, we aim to complement the scarce available data on Italian young populations regarding exclusively sleep habits, circadian preference, [15,16], and the presence of specific sleep problems [19,20,21].

Specifically, our objectives are as follows: (a) to determine the prevalence of good and bad sleepers in a sample of Italian children, preadolescents, and adolescents; (b) to investigate determinants of sleep quality perception among a wide repertoire of variables including sleep habits, sleep features, sleep–wake behavioral problems, circadian preference, and dream recall frequency and quality; (c) to explore whether these subjective predictors of sleep quality perception change across age ranges.

## 2. Materials and Methods

### 2.1. Participants and Procedure

A survey has been carried out in public primary and middle lower schools located in Northern and Central Italy: Como, Sansepolcro (Arezzo), Cospaia (Perugia), Selci (Rieti), San Giustino (Perugia), and Lama (Siena). These schools were randomly selected from the list of all public primary and middle lower schools of these Italian cities. Headmasters of the selected schools were first contacted through a formal letter, introducing the research and the professionals involved. Then, all the procedures, instruments, and aims of the study were explained in a meeting, extended to teachers and parents’ representatives. A further meeting served to illustrate the study procedure in detail to all children’s parents and to collect their informed consent.

One thousand four hundred thirty students (731 males, 699 females) were recruited for the study (6–8 years: *n* = 307, 164 males, 143 females; 9–11 years: *n* = 717, 373 males, 344 females; 12–14 years: *n* = 406, 194 males, 212 females; see Table 1). The only exclusion criterion was the presence of a diagnosed cognitive or learning disorder.

The study design was submitted to the Ethical Committee of the Department of Psychology, University of Campania “L. Vanvitelli”, which approved the research (code 22/2020) and certified that the involvement of human participants was performed according to acceptable standards.

### 2.2. Instruments

Data were collected through the School Sleep Habits Survey [50,51] in its Italian version [15,16]. The questionnaire addresses the following areas:

(a) Sleep habits, composed of six open questions assessing habitual bedtime, rise time, and sleep duration, in hours and minutes, on both schooldays and weekends;
(b) Sleep features, including one question on habitual sleep latency, consisting of a single forced-choice item with six response categories (“0 to 5 min”, “6 to 15 min”, “16 to 30 min”, “31 to 45 min”, “46 to 60 min”, and “more than one hour”); a forced question examining whether sleep duration is considered sufficient (“How often do you think you sleep enough?”), with five choices (“always”, “often”, “sometimes”, “seldom”, and “never”); a forced question investigating the frequency of nocturnal awakenings (“Never”, “Once”, “2 or 3 times”, “More than 3 times”, and “I have no idea”); a forced question evaluating daytime napping (“Some people take naps in the daytime every day, others never do. When do you nap?”), with four choices (“I never nap”; “I sometimes nap on school days”; “I sometimes nap on weekends”; “I never nap unless I am sick”);
(c) Daytime behavior and sleep disturbances (DBSD), which includes three scales: a sleepiness scale (SLS), a depressed mood scale (DMS) and a sleep–wake problems behavior scale (SWP). The SLS is composed of nine items assessing the ease of staying awake in different situations (“talking vis a vis with someone else”, “travelling on public transports”, “watching a show”, “watching television or listening to music”, “reading or studying”, “during a school test”, “sitting in class”, “working at the computer”, and “playing a videogame”): respondents had to choose among four ordinal alternatives, ranging from “no difficulty staying awake” to “struggling to stay awake but falling asleep”. A global vigilance score, ranging from 9 to 36, was then obtained by summing up scores at all of the nine items, with higher scores reflecting higher sleepiness levels. The DMS consists of 5 multiple choice items assessing the presence of depressed mood over the last two weeks. Participants have to choose among three alternatives (“not at all”; “somewhat”; “much”). A global index of depressed mood, ranging from 10 to 30, is obtained by averaging scores and multiplying them by ten, with higher scores indicating more depressed mood. Finally, the SWP is made up of 10 items, assessing how often the subjects have experienced some sleep/wake-related perceptions and problems over the last two weeks (“being late at school for having slept too long”, “falling asleep in a morning class”, “falling asleep in an evening class”, “going to bed late in the evening”, “staying awake all night long”, “sleeping until noon”, “having difficulties waking up in the morning”, “having problems falling asleep at bedtime”, “having nightmares”, and “going to bed too early because of excessive sleepiness”). The answers are graded on a five-point scale, ranging from “never” to “always”. The total score, ranging from 10 to 50, is computed by summing the answers to all items, with higher scores reflecting more sleep-related problems;
(d) Circadian preference, assessed by means of a Morningness–Eveningness Questionnaire (MEQ), composed of ten items. The global score, ranging from 43 (extreme morningness) to 10 (extreme eveningness), allows to identify subjects as morning types (M-types), intermediate types (I-types), and evening types (E-types);
(e) Sleep quality: an item about sleep quality perception (“Do you consider yourself as a good or a bad sleeper?”) was added to the original Italian version of the questionnaire [16]. Subjects had to choose among two response alternatives (“a good sleeper”, “a bad sleeper”);
(f) Dreaming: two questions about dreams were also added to the original questionnaire [16]: one regarding dream recall frequency (“How often you remember having dreamed?”), with four ordinal alternatives ranging from “always” to “never”, and one regarding dream pleasantness (“How are your dreams usually?”), with five forced-choice alternatives ranging from “wonderful and exciting” to “horrible and frightening”.

### 2.3. Procedure

Questionnaires were administered by a trained experimenter during school hours in presence of the teachers. Participants filled them out individually. In view of the subjects’ young age, the experimenter remained in the classroom throughout the administration procedure, being available to answer any question arising during questionnaire completion. He was specifically instructed to provide standardized answers, which included question rephrasing and examples.

### 2.4. Data Analysis

After descriptive statistics, the global sample was split in two groups (“good sleepers” and “bad sleepers”), based on the answer to the question assessing overall subjective sleep quality. The prevalence of good and bad sleepers was calculated for the total sample, for males and females separately, and within three different age groups [52,53]: children (6–8 years), preadolescents (9–12 years), and adolescents (12–14 years).

Good and bad sleepers were then compared for the following dependent variables:

- sleep habits, that is, sleep duration in minutes, bedtime and rise time—all variables reported for schooldays and weekends—plus the differences (Δ) between schooldays (SD) and weekends (WE) in bedtime (reported as “Δ-SD/WE bedtime”), rise time (indicated as “Δ-SD/WE rise time”), and sleep duration (reported as “Δ-SD/WE sleep duration”);
- habitual sleep latency;
- sufficiency of sleep;
- night awakenings frequency;
- daytime napping frequency;
- sleepiness global score, obtained from the sleepiness scale;
- depressive mood index, calculated from the depressed mood scale;
- sleep–wake behavioral problems global score, obtained from sleep–wake problems behavior scale;
- circadian preference, assessed through the morningness–eveningness questionnaire;
- dream frequency and dream pleasantness.

For comparisons, nonparametric Mann–Whitney U-test was used for all cardinal variables. Chi-square test was carried for all binomial variables. Subjective sleep quality determinants were assessed through a stepwise logistic regression, with sleep quality perception (“good sleep” and “bad sleep”) as dependent variable and all other variables (Δ-SD/WE of bedtime; rise time and sleep duration; habitual sleep latency; sufficiency of sleep; night-awakenings frequency; daytime napping frequency; the sleepiness global score; the depressive mood index; the sleep–wake behavioral problems global score; morningness–eveningness global score; frequency and pleasantness of dreams) as independent variables.

To correct for multiple testing without running a too high risk of Type II Error (see, for example, [54]), the conventional alpha value (*p* ≤ 0.05) was divided by five, that is, by the number of relevant “dimensions” addressed in our research (“sleep habits”, “sleep features”, “daytime behavior and sleep disturbances”, “circadian preference”, and “dreaming”)**.** Therefore, significance was set at *p* ≤ 0.01.

Since we employed nonparametric statistical tests, following Lehmann [55], we computed the sample size required for a parametric test and added 15%. Given the unequal distribution of bad and good sleepers in the population, with an alpha value of 0.05, a sample of at least 300 participants was required to detect medium effects (d = 0.5) with a power of 0.80 [56]. On this basis, we recruited participants in the mentioned schools until reaching the minimum value of at least 300 in each group. Given the constraint of including the entire student population of each school and the uneven distributions of classes, the 9–11 age-group had a higher number of participants.

## 3. Results

### 3.1. Response Rate

The questionnaire was completed by all children (N = 1430). However, eight questionnaires were excluded due to aberrant values, and nine subjects did not answer the question about global sleep quality. Thus, the final sample for data analysis included 1413 subjects (6–8 years, *n* = 302, 160 males, 142 females; 9–11 years, *n* = 705, 364 males, 341 females; 12–14 years, *n* = 406, 194 males, 212 females).

### 3.2. Overall Sleep Quality

To the question about global sleep quality, 167 subjects answered to be “bad sleepers” (11.7% of the total sample), whereas 1246 answered to be “good sleepers” (87.6% of the total sample).

**Figure 1:**
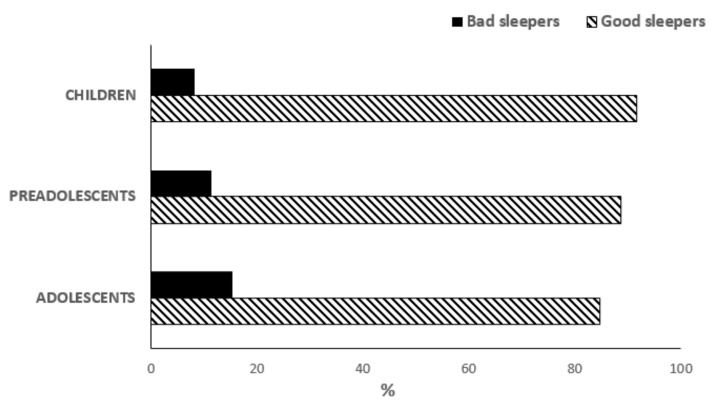
Prevalence of good and bad sleepers in children, preadolescents and adolescents.

The percentage of bad sleepers differed significantly across age ranges (Figure 1; 6–8 years: 8.3%; 9–11 years: 11.3%; 12–14 years: 15.3%; χ^2^ = 8.4, *p* = 0.015) but not between genders either in the total sample (M: 7.3%, F: 6.5%, χ^2^ = 0.4, *p* = 0.851) or in the three age groups (6–8 years: M = 5.3%, F = 3.0%, χ^2^ = 1.3, *p* = 0.249; 9–11 years: M = 6.2%, F= 5.1%, χ^2^ = 0.4, *p* = 0.522; 12–14 years: M = 6.4%, F = 8.9%, χ^2^ = 1.0, *p* = 0.317).

### 3.3. Sleep Habits

Table 2 displays comparisons between good and bad sleepers in sleep habits variables, separately for each age group (Table 2). No significant between-groups differences were found in bedtime, rise time, and sleep duration on schooldays or weekends in either of the three age groups. Similarly, Δ-SD/WE of bedtime, rise time, and sleep duration did not show differences between good and bad sleepers in children, preadolescents, or adolescents.

### 3.4. Sleep Features

Table 3 displays comparisons between good and bad sleepers in sleep features, separately for each age group (Table 3). In all age groups, bad sleepers referred to a longer sleep onset latency and more frequently reported insufficient sleep and nocturnal awakening compared with good sleepers.

In three age groups, no significant differences emerged between good and bad sleepers in daytime napping frequency.

### 3.5. Daytime Behaviour and Sleep Disturbances

A significant difference emerged between bad and good sleepers at the global score of sleepiness scale and sleep–wake behavioral problems in all age groups (Table 4). Moreover, in the three age groups, bad sleepers showed higher indices in depressive mood scale (Table 4).

### 3.6. Circadian Preference

**Figure 2:**
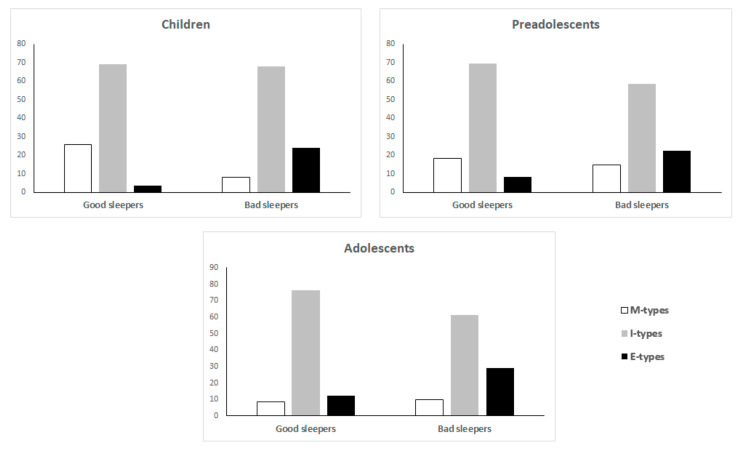
Frequency distribution of morning types (M-types), intermediate types (I-types) and evening types (E-types) in good and bad sleepers in children, preadolescents, and adolescents.

Frequency distribution of M-types, I-types and E-types differed between good and bad sleepers in children (χ^2^= 20.8, *p* < 0.001), preadolescents (χ^2^= 16.1, *p* = 0.002) and adolescents (χ^2^ = 11, *p* = 0.004), with bad sleepers more frequently displaying an evening preference compared with good sleepers (Figure 2).

In addition, significant differences were found in Morningness-Eveningness Questionnaire (MEQ) global scores between good and bad sleepers in all age groups. Specifically, bad sleepers reported lower scores, indicating a higher degree of eveningness (children: good sleepers mean = 30.5 ± 4.6, bad sleepers mean = 26.2 ± 5.8; U = 1914, *p* < 0.001; preadolescents: good sleepers mean = 29.2 ± 4.9, bad sleepers mean = 26.4 ± 5.9; U = 16957, *p* < 0.001; adolescents: good sleepers mean = 28.0 ± 4.6, bad sleepers mean = 24.8 ± 6.1; U = 6858, *p* < 0.001).

### 3.7. Dreaming

In all three age groups, bad and good sleepers reported the same dream frequency (median = 3, that is, “sometimes”; children: U = 3115, *p* = 0.391; preadolescents: U = 4182, *p* = 0.638; adolescents: U = 10351, *p* = 0.702).

Instead, dreams appeared more unpleasant in bad sleepers of all age groups (children: good sleepers median = 2, that is, “Nice, funny”, bad sleepers median = 5, that is, “horrible and frightening”; U = 1939, *p* < 0.01; preadolescents: good sleepers median = 2, bad sleepers median = 3, that is, “So and so, not very interesting”; U = 16566, *p* < 0.001; adolescents: good sleepers median = 2, bad sleepers median = 3; U = 8704, *p* = 0.016).

### 3.8. Subjective Sleep Quality Determinants

Different sleep quality determinants emerged in the three age groups. In children (pseudoR2 = 0.4), sleep quality was predicted by depressed mood (*p* < 0.001), morningness–eveningness global score (*p* = 0.003), and pleasantness of dreams (unpleasant dreams, *p* = 0.01); in preadolescents (pseudoR^2^ = 0.3), by sleep latency (*p* = 0.007), awakenings frequency (*p* = 0.003), sufficiency of sleep (*p* = 0.001), depressed mood (*p* < 0.001), and unpleasant dreams (*p* = 0.002); in adolescents (pseudoR^2^ = 0.4), by awakenings frequency (*p* < 0.001), sufficiency of sleep (*p* < 0.001), and depressed mood (*p* < 0.001).

## 4. Discussion

To our knowledge, this study is the first to investigate children’s and adolescents’ sleep quality perception through a direct question: “Do you consider yourself as a good or a bad sleeper?”. In fact, the majority of studies evaluating sleep quality at young ages referred to nonspecific variables such as presence of sleeping problems or sleep complaints [13,31,57,58]; in addition, when surveys were performed on children younger than 10 years, the sleep quality assessment was based on parents’ and teachers’ reports, which increases the risk of low accuracy [59,60,61].

A first remark concerns the observed proportion of subjects considering themselves as bad sleepers, which is quite low in children (8.3%) and increases across age groups, reaching 15.3% at 12–14 years. This percentage is in line with data reported by Manni and colleagues [22] in 17-year-old Italian students and with those reported in other countries among teenagers [62]. Interestingly, no difference in the proportion of good and bad sleepers was found between males and females. This result is in accordance with data showing no gender differences in the frequency of specific sleep problems in children [63] and in global scores at the sleep–wake problems behavior scale in subjects aged 8–14 years [16]. In contrast, other studies conducted on children [64], adolescents [22,65], and adults [66,67,68] show a greater number of sleep complaints in females compared with males. Possibly, the gender disparity in sleep quality occurs only from late adolescence.

A major result of our study is the profile of self-reported sleep features identifying the “bad sleeper” across age groups. Indeed, good and bad sleepers did not differ in either of the three age groups in sleep habits (bed and rise times, etc.). Instead, longer sleep onset latency, more frequent insufficient sleep, and nocturnal awakenings were reported by bad sleepers compared with good sleepers at all ages. Interestingly, these self-reported sleep features correspond to those that are commonly considered, in literature, based on objective sleep measures, as signs of poor sleep in children and adolescents [13,18,31,57,58,69].

Bad sleepers in all age groups also displayed higher scores in the sleepiness scale, sleep–wake problems behavior scale, and depressive mood scale compared with good sleepers. The higher sleepiness found in bad sleepers is not surprising considering the negative influence of poor sleep on diurnal vigilance levels commonly reported in literature [70,71]. This result also confirms daytime sleepiness as one of the most common complaints associated to sleep problems in children and adolescents [72,73]. As for depression, our finding is in line with data by Lovato and Gradisar [74], who reported poor sleep quality, in terms of sleep efficiency and wake after sleep onset, as a precursor in the development of depression in adolescents; furthermore, Chiu and colleagues [75] found a dose–response association between sleep duration and suicidality in the same age group. Instead, data on the relationships between sleep features and depressive symptoms in children are still inconsistent [76].

As for chronotypology, bad sleepers showed greater evening preference than good sleepers independently of age, in line with previous data showing higher prevalence of sleep complaints in evening types in both children and adolescents [18]. Furthermore, adolescent evening types have been found to complain of daytime sleepiness and to refer attention problems to a greater extent than other chronotypes [15], suggesting that the lower daytime vigilance levels reported by bad sleepers could also be modulated by chronotypology in addition to poor sleep quality. Indeed, with increasing age, children tend to go to bed later, have an increased preference for evening activities, and sleep less. This behavior change is driven by external factors (e.g., increased pressures from academic, social, and extracurricular activities) as well as biological circadian factors and may result in a “social jet lag” (i.e., a misalignment between individual biological rhythms and the social rhythm imposed by school schedules [77]). This mismatch leads many adolescents to accumulate a significant sleep debt, especially during the school week, and to feel sleepy during the day [73].

Of note, the emotional valence of mental activity during sleep also appears linked to sleep quality perception. Actually, in all age groups, bad sleepers reported unpleasant dreams more frequently than good sleepers, whereas there were no differences between groups in dream recall frequency. This result suggests the existence of a relationship between dream and sleep satisfaction, in line with previous work showing a detrimental effect of negatively toned dreams on sleep quality [78,79,80]. Moreover, it is in accordance with a recent study showing that children consider nightmares as a potential cause of bad sleep [48].

The second bulk of results of our study regards the subjective determinants of sleep quality perception. Our findings show that predictors of sleep quality satisfaction change across ages. In children, poor subjective sleep quality appears to be mainly determined by psychophysiological features related to daytime, for example, depressed mood, unpleasant dream recall, and chronotypology, rather than sleep features per se. This result suggests that, in children, the presence during wake of emotional problems such as depressed mood and recall of unpleasant dreams may lead subjects to retrospectively define sleep quality as poor and unsatisfactory (an idea proposed, in adults, by [81,82,83]). At later ages, instead, sleep features, such as longer sleep latency, more frequent awakenings, and insufficient sleep, add to the former determinants. A twofold hypothesis may be proposed to explain this finding. On one side, sleep features may appear as determinants of bad sleep perception as a result of the occurrence, with increasing age, of actual objective sleep modifications, such as sleep onset delay, decreased nocturnal sleep time, increased arousal frequency, and wake after sleep onset [84,85]. On the other hand, preadolescents and adolescents could pay greater attention to their own sleep characteristics than children, in the same manner as they do with their body changes occurring during this period of development [86,87,88,89].

As for comparisons with the adult population, determinants of poor sleep perception appear to partially overlap. Indeed, the sleep features we found to predict sleep perception among preadolescents and adolescents, that is, ease of falling asleep, frequency of awakening, and sleep continuity, are similar to subjective sleep quality determinants described in adults [32,49]. However, other sleep characteristics, such as calmness of sleep and freshness after awakening [42,49], reported in adults and in older populations, probably determine sleep quality perception starting from later ages.

### Limitations

Our results should be considered in light of some limitations to be overcome in future research. In this study, only subjective data were collected. Indeed, polysomnographic recordings over repeated consecutive nights are warranted to complement our data, with the aim to evaluate the accordance between objective and subjective sleep features as well as to explore whether the presence of daytime emotional and behavioral problems may depend on objective sleep impairments. In fact, as suggested in previous studies [34,81,90], a cognitive bias may contribute to retrospectively define sleep quality as poor and unsatisfactory. Furthermore, in our sample, we cannot exclude the influence of inadequate pre-bed activities, such as the use of electronic devices, since prior studies showed a detrimental effect on sleep quality [91,92]. Finally, it should be acknowledged that determinants of sleep satisfaction in children and adolescents could be culturally mediated, given the relevant differences in sleep habits and features observed between different sociocultural contexts [18].

## 5. Conclusions

In conclusion, our study showed that the perception of poor sleep at young ages is associated with certain self-reported sleep characteristics (i.e., long sleep latencies, frequent night awakenings, insufficient sleep, evening circadian preference) that appear stable across different age groups (from 6 to 12 years). Instead, subjective sleep variables predicting bad sleep perception differ as a function of age. Cognitive and emotional development occurring from childhood to adolescence might be responsible for these differences and they should be taken into account in sleep quality assessments at young ages. Moreover, knowing which self-reported sleep characteristics and daytime psychological features are associated to a subjective bad sleep perception may be of clinical interest: it may contribute to precociously detect those subjects who might be more vulnerable than others to develop sleep problems in the future, arising also the possibility to plan specific interventions in order to prevent psychological disturbances and sleep disorders among adolescents.

## Figures and Tables

**Table 1:** Demographic characteristics of the sample (N= total number of participants; M= males; F= females).

	*N*	M	F	6–8	9–11	12–14
Como	736	377	359	153	362	221
Sansepolcro	534	268	266	130	218	185
Cospaia	47	33	14	12	35	0
Lama	6	1	5	2	4	0
Selci	34	18	16	1	33	0
Sangiustino	73	34	39	9	65	0
Total sample	1430	731	699	307	717	406

**Table 2:** Descriptive data on sleep habits and their comparison between good and bad sleepers in children, preadolescents and adolescents (SD= standard deviation; U = Mann Whitney test; Δ-SD/WE Bedtime = difference between schooldays and weekends in bedtime; Δ-SD/WE Rise time = difference between schooldays and weekends in rise time; Δ-SD/WE sleep duration = difference between schooldays and weekends in sleep duration).

Sleep Habits	Good Sleepers	Bad Sleepers	U	*p*
	Mean *±* SD	Mean *±* SD		
Children				
Bedtime schooldays	21:24 ± 00:38	21:29 ± 00:33	3017	0.298
Bedtime weekend	22:30 ± 00:48	22:35 ± 00:55	3040	0.387
Rise time schooldays	07:04 ± 00:25	07:05 ± 00:27	3411	0.972
Rise time weekend	09:14 ± 01:13	09:00 ± 01:34	2980	0.303
Sleep duration schooldays	09:25 ± 00:53	09:01 ± 01:01	2645	0.046
Sleep duration weekend	10:32 ± 01:13	09:52 ± 01:19	2642	0.053
Δ-SD/WE Bedtime	01:09 ± 00:43	01:10 ± 00:38	3263	0.756
Δ-SD/WE Rise time	02:11 ± 00:14	01:54 ± 01:30	2971	0.308
Δ-SD/WE Sleep duration	01:23 ± 01:05	01:17 ± 00:57	3405	0.938
Preadolescents				
Bedtime schooldays	21:45 ± 00:42	21:49 ± 00:49	24784	0.917
Bedtime weekend	22:55 ± 01:14	22:59 ± 00:56	24029	0.766
Rise time schooldays	07:06 ± 00:22	07:03 ± 00:28	22852	0.205
Rise time weekend	09:22 ± 01:15	09:05 ± 01:10	21853	0.112
Sleep duration schooldays	09:15 ± 01:01	09:04 ± 01:09	22469	0.206
Sleep duration weekend	10:17 ± 01:19	09:51 ± 01:32	20961	0.047
Δ-SD/WE Bedtime	01:17 ± 01:10	01:15 ± 00:47	24305	0.912
Δ-SD/WE Rise time	02:15 ± 01:16	02:02 ± 01:10	22330	0.193
Δ-SD/WE Sleep duration	01:23 ± 01:40	01:24 ± 01:30	23984	0.852
Adolescents				
Bedtime schooldays	22:19 ± 00:59	22:25 ± 00:38	10051	0.487
Bedtime weekend	23:38 ± 00:53	23:51 ± 00:50	8869	0.060
Rise time schooldays	07:01 ± 00:19	06:57 ± 00:27	9758	0.285
Rise time weekend	09:43 ± 01:15	09:45 ± 01:33	10496	0.958
Sleep duration schooldays	08:30 ± 00:58	08:12 ± 00:56	8712	0.023
Sleep duration weekend	09:55 ± 01:21	09:52 ± 01:31	10328	0.772
Δ-SD/WE Bedtime	01:22 ± 01:27	01:26 ± 00:48	9290	0.158
Δ-SD/WE Rise time	02:41 ±01:15	02:47 ± 01:34	10424	0.890
Δ-SD/WE Sleep duration	01:37 ± 01:07	01:51 ± 01:10	9400	0.164

**Table 3:** Median, mean, and standard deviation of sleep features and their comparison between good and bad sleepers in children, preadolescents, and adolescents. Mean and standard deviation are in brackets.

Sleep Features	Good Sleepers	Bad Sleepers	U	*p*
Children				
Habitual sleep latency	2 (2.1 ± 1.3)	2 (2.8 ± 1.7)	2612	0.042
Sufficiency of sleep	2 (2.1 ± 1.2)	3 (2.7 ± 1.3)	2497	0.016
Nocturnal awakening frequency	2 (1.9 ± 1.2)	2 (2.6 ± 1.3)	2354	0.004
Nap frequency	1 (1.9 ± 1.2)	2 (2.1 ± 1.2)	3060	0.308
Preadolescents				
Habitual sleep latency	2 (2.5 ± 1.4)	3 (3.3 ± 1.7)	16892	<0.001
Sufficiency of sleep	2 (2.2 ± 1.0)	3 (2.8 ± 1.1)	15960	<0.001
Nocturnal awakening frequency	2 (2.1 ± 1.2)	3 (2.9 ± 1.4)	15348	<0.001
Nap frequency	1 (1.9 ± 1.2)	2 (2.1 ± 1.3)	22438	0.113
Adolescents				
Habitual sleep latency	2 (2.5 ± 1.3)	3 (3.1 ± 1.7)	7620	<0.001
Sufficiency of sleep	2 (2.3 ± 0.9)	3 (3.3 ± 0.9)	4679	<0.001
Nocturnal awakening frequency	2 (1.9 ± 1.1)	3 (2.8 ± 1.1)	5877	<0.001
Nap frequency	1 (1.9 ± 1.2)	2 (1.9 ± 1.1)	10072	0.47

**Table 4:** Comparisons between good and bad sleepers in daytime behavior and sleep disturbances *(*DBSD) within children, preadolescents and adolescents groups. SLS = sleepiness scale global score; SWP = sleep–wake behavioral problems global score; DMS = depressive mood scale index.

Age Groups	DBSD	Good Sleepers	Bad Sleepers	U	*p*
		Mean *±* SD	Mean *±* SD		
Children	SLS	12.1 ± 2.5	15.4 ± 4.5	1817	>0.001
	SWP	19.1 ± 5.5	23.4 ± 5.4	1612	0.001
	DMS	14.8 ± 3.5	19.6 ± 3.5	1473	>0.001
Preadolescents	SLS	12.5 ± 2.9	13.9 ± 4.4	20527	0.014
	SWP	20.3 ± 5.4	23 ± 5.7	16029	>0.001
	DMS	15.3 ± 3.3	18.7 ± 3.7	12551	>0.001
Adolescents	SLS	12.3 ± 2.6	13.9 ± 3.3	7304	>0.001
	SWP	20.5 ± 5.6	25.0 ± 4.8	5151	>0.001
	DMS	16.4 ± 3.6	19.6 ± 3.6	5584	>0.001
